# New Insights Into an Old Vaccine: Potency and Breadth of Neutralizing Antibodies Elicited by Yellow Fever Vaccine 17D Are Boosted by Heterologous *Orthoflavivirus* Infection

**DOI:** 10.1093/infdis/jiag077

**Published:** 2026-02-11

**Authors:** Felicity J Coulter, Courtney A Micheletti, Abram E Estrada, Shuhua Luo, Samantha R Osman, Chad D Nix, Sophia Y Hu, Sarah M Sampouw, Bettie W Kareko, Allison L Naleway, William B Messer

**Affiliations:** Department of Molecular Microbiology and Immunology, Oregon Health & Science University, Portland, Oregon, USA; Department of Molecular Microbiology and Immunology, Oregon Health & Science University, Portland, Oregon, USA; Department of Molecular Microbiology and Immunology, Oregon Health & Science University, Portland, Oregon, USA; Department of Molecular Microbiology and Immunology, Oregon Health & Science University, Portland, Oregon, USA; Department of Molecular Microbiology and Immunology, Oregon Health & Science University, Portland, Oregon, USA; Department of Molecular Microbiology and Immunology, Oregon Health & Science University, Portland, Oregon, USA; Department of Molecular Microbiology and Immunology, Oregon Health & Science University, Portland, Oregon, USA; Department of Molecular Microbiology and Immunology, Oregon Health & Science University, Portland, Oregon, USA; Department of Molecular Microbiology and Immunology, Oregon Health & Science University, Portland, Oregon, USA; Kaiser Permanente Center for Health Research, Portland, Oregon, USA; Department of Molecular Microbiology and Immunology, Oregon Health & Science University, Portland, Oregon, USA; Division of Infectious Diseases, Department of Medicine, Oregon Health & Science University, Portland, Oregon, USA; Program in Epidemiology, Oregon Health & Science University–Portland State University School of Public Health, Portland, Oregon, USA

**Keywords:** 17D, neutralizing antibodies, *Orthoflavivirus*, yellow fever virus

## Abstract

**Background:**

Yellow fever vaccine 17D has controlled yellow fever for almost 90 years and is considered highly successful. However, with recent outbreaks in South America and Africa, yellow fever continues to represent a significant threat to public and global health. Despite the long-standing success, few studies have characterized the capacity of 17D-immune sera to neutralize wild type (WT) yellow fever viruses (YFVs).

**Methods:**

Using 17D and 13 WT YFVs, we conducted focus neutralization tests against sera from a unique cohort of 36 nonendemic vaccinees with diverse *Orthoflavivirus* histories. By using WT YFVs that represent the known YFV genotypes, we characterized the potency and breadth of 17D-elicited serum neutralizing antibodies against these WT YFVs in the context of heterologous *Orthoflavivirus* infection.

**Results:**

We found significant variability in neutralization test titers and rates of seropositivity of 17D-immune sera across all viruses and specifically against South America genotype I isolates. Vaccinee sera with serologic evidence of heterologous *Orthoflavivirus* infection had significantly greater potency against South America genotype I strains as compared with sera without evidence of *Orthoflavivirus* infection, suggesting a boosting effect from heterologous *Orthoflavivirus* immunity.

**Conclusions:**

These results add significantly to what is known regarding the WT YFV antigenic immune landscape, including novel insights into the role of heterologous *Orthoflavivirus* infection in 17D immunity that have the potential to improve future vaccine design and deployment strategies.

## Teaser

The ability of neutralizing antibodies elicited by the yellow fever virus vaccine 17D to neutralize WT YFV is incompletely characterized. We report variable neutralization of WT YFV strains and enhanced potency in vaccinees with heterologous *Orthoflavivirus* immunity, informing future vaccine design and deployment strategies.

**(See the Editorial Commentary by Bailey and Stapleton on pages e216–8.)**

Mosquito-borne yellow fever virus (YFV) is the prototypic *Orthoflavivirus* and cause of yellow fever, with up to 173 000 severe infections and 82 000 deaths annually across Africa and South America [1]. Live-attenuated yellow fever vaccine 17D, developed in the 1930s, plays an essential role in controlling disease, preventing up to 119 000 cases annually [1]. 17D is highly immunogenic, eliciting serum neutralizing antibodies (NAbs), a widely accepted correlate of protection [2, 3], in >95% of vaccinees by 30 days postvaccination [4, 5]. The gold standard for characterizing 17D-elicited serum NAb titers is the plaque reduction neutralization test, most using the 17D strain [6–8] as the test virus. Crucially, these 17D-based plaque reduction neutralization test data played a key role shaping recommendations [10–14] despite limited knowledge regarding the capacity of 17D-elicited NAbs to neutralize most wild type (WT) strains.

There are 7 WT YFV genotypes: West Africa I; West Africa II, which includes the 17D parental strain Asibi; East Africa; East/Central Africa; Angola; South America (SA) I and SA-II [9]. Phylogenetic analyses estimate that South American strains diverged from West African strains almost 500 years ago following presumed introduction into the Americas via the slave trade [10].

The potency of 17D-immune sera against WT viruses vs 17D has undergone limited evaluation to date: Haslwanter et al [11] and Goncalves et al [12] found that the potency of 17D-immune sera against SA-I strains among vaccinees is significantly reduced against SA-I as compared with 17D and Asibi. While Haslwanter et al identified specific amino acid polymorphisms that accounted for reduced potency of 17D-immune sera against SA-I viruses, the potency and breadth of 17D-immune sera against the full antigenic landscape of WT YFV remain largely unexamined.

To address this knowledge gap, we assembled a panel of 13 WT YFVs representing all 7 genotypes and immune sera from a cohort of nonendemic 17D vaccinees with diverse *Orthoflavivirus* infection histories. We then comprehensively evaluated the potency and breadth of 17D-elicited serum against the full YFV antigenic landscape, providing the most extensive characterization of YFV potency and breadth to date, including a novel potential role for heterologous *Orthoflavivirus* infection in 17D immunity.

## METHODS

### Human Research Ethics

This study was approved by the Oregon Health & Science University (OHSU) Institutional Review Board (10212 and 20910) with the Kaiser Pacific Northwest Center for Health Research ceding oversight to OHSU.

### Study Participants

All participants were recruited from the Portland area, Oregon, based on prior suspected or confirmed *Orthoflavivirus* infection with and without history of vaccination with 17D [13].

### Serum

Serum was collected in serum separator tubes, allowed to clot, centrifuged (1000*g* × 10 minutes), and stored at −20 or −80 °C until use. Sera were heat inactivated at 56 °C for 30 minutes prior to neutralization assays. *Orthoflavivirus* infection against dengue virus 1 to 4 (DENV-1 to DENV-4) and Zika virus (ZIKV) was characterized by a focus reduction neutralization test (FRNT). A 50% FRNT (FRNT_50_ or NT_50_) >1:20 in the setting of a clinical history of DENV or ZIKV infection was considered seropositive.

### Viruses

WT YFVs were obtained from the US Centers for Disease Control and Prevention, the World Reference Center for Emerging Viruses and Arboviruses, and Betânia Drumond from the Universidade Federal de Minas Gerais (Supplementary Table 1). 17D was propagated from a vial of YF-VAX.

### Virus Sequencing

Viral RNA was extracted by the Quick-RNA Viral Kit (catalog R1034; Zymo Research Corporation), and complementary DNA was synthesized by a SuperScript III First-Strand Synthesis System (Thermo Fisher) and primer YFVAmp2A (Supplementary Table 2). Polymerase chain reaction amplification was performed with Phusion High-Fidelity DNA Polymerase (New England Biolabs) and primers YFVAmp1A and YFVAmp2A (Supplementary Table 2). Polymerase chain reaction products were visualized on 1% agarose gel with SYBR Safe DNA gel stain (Thermo Fisher), and the target band (3.5 kb) was excised and purified with the QIAquick Gel Extraction Kit (Qiagen). Purified products were sequenced commercially (Genewiz), using primers specified in Supplementary Table 2. Contigs from .ab1 files were aligned and assembled using Geneious Prime (version 2024.0.7).

Phylogenetic analysis was conducted in MEGA11 [14, 15]. The evolutionary history was inferred from the full-length E protein sequence (493 residues) by the maximum likelihood method and Dayhoff matrix-based model [16].

### Virus Propagation

Viruses were propagated at biosafety level 3 (WT) or biosafety level 2 (17D) in Vero E6 cells (CRL-1586; ATCC) transfected with a plasmid encoding human furin, although furin was not overexpressed in these cells. T-75 tissue culture flasks (Fisherbrand) were seeded with 5.0E + 06 cells in 12 mL of media—MEM/EBSS (HyClone), 1% v/v nonessential amino acids (NEAA; Gibco), 1% v/v antibiotic-antimycotic (Gibco), 10% v/v heat-inactivated fetal bovine serum (Avantor), and 460-µg/mL G418 (Thermo)—and incubated at 37 °C, 5% CO_2_. Near confluent flasks were washed with phosphate-buffered saline (PBS; HyClone). Virus was thawed or reconstituted from lyophilized cultures with dilution media (DM; MEM/EBSS, 1% v/v NEAA, 1% v/v antibiotic-antimycotic, 2% v/v heat-inactivated fetal bovine serum), added to flasks in DM, and incubated as previously indicated for 1 hour. The inoculum was then discarded, and Vero complete media was added (MEM/EBSS with 10% fetal bovine serum, anti-anti, L-glutamine). Tissue culture supernatants were harvested when the cytopathic effect was ≥40%. Tissue culture supernatants were clarified (1000*g* × 10 minutes), resuspended in 10% v/v sucrose phosphate glutamate buffer (2.18M sucrose, 38mM KH_2_PO_4_, 72mM K_2_HPO4, 60mM L-glutamic acid), and stored at −80 °C for ≥48 hours before use. All assays were performed via passage 2 virus stocks.

### YFV Focus Reduction Neutralization Test

Vero cells were seeded in 96-well plates at 2.0E + 04 cells per well in Vero complete media and incubated overnight at 37 °C, 5% CO_2_. Participant serum was diluted 4-fold in DM from 1:5 to 1:1280. Biological duplicates of serum dilutions were prepared for each virus, with a virus-only control well per serum replicate. Viruses were thawed and diluted in DM to 40 to 150 foci per well. Equal volumes of virus and diluted serum were combined to give final serum dilutions of 1:10 through 1:2560 and incubated for ∼60 minutes at 37 °C, 5% CO_2_. These were then added to previously seeded 96-well plates and incubated for 1 hour at 37 °C, 5% CO_2_; after which, they were overlaid with OptiMEM (Gibco) supplemented with 1% NEAA, 1% antibiotic-antimycotic, 2% v/v fetal bovine serum, and 5 g of methylcellulose (Sigma-Aldrich). Following a 48- to 68-hour incubation, overlay media was removed, and plates were washed with PBS, fixed with 4% paraformaldehyde (Sigma-Aldrich), and incubated overnight at 4 °C under blocking buffer (BB; PBS with 2% heat-inactivated normal goat serum [Rockland Immunochemicals Inc] and 0.4% Triton-X). Plates were washed with PBS; 4G2 primary antibody (1 mg/mL, HB-112; ATCC) diluted 1:750 in BB was then added; and plates were incubated overnight at 4 °C. Plates were washed with PBS and incubated for 45 to 60 minutes with goat–anti-mouse horseradish peroxidase–conjugated IgG antibody (BioLegend) diluted 1:1000 in BB; they were then washed 3 times with PBS before TrueBlue Peroxidase Substrate (Kirkegaard & Perry Laboratories Inc) was added to each well. Plates were developed until foci were visible, quenched with water, and air-dried. Foci were counted with CTL ImmunoSpot 7.0.26.0 (Cellular Technology LTD). FRNT values were calculated in Prism (version 10.3.0; GraphPad) with an ECAnything 4-parameter sigmoidal dose-response curve, with lower and upper limits set at 0 and 100. DENV and ZIKV FRNTs were similarly performed in a 24- or 96-well format with infection incubations of 24 hours (ZIKV) and 72 hours (DENV), as previously described [13, 17].

### Antigenic Cartography

Antigenic maps were constructed as previously described [17–19] using NT_50_ data in the Racmacs package (version 4.3.1) in RStudio (version 2023.06.1 + 524).

### Statistical Methods

Nonparametric methods were used for FRNT titer comparisons. Friedman test with Dunn multiple pairwise comparison was used to compare FRNT titers; χ^2^ test was used to compare the proportion of seropositive and seronegative vaccinees for each virus; and Mann-Whitney *U* test was used to compare FRNT titers between 17D-only and heterologous vaccinees for each virus. Dayhoff values were evaluated by a Spearman rank correlation with Pearson correlation applied as an indicator of fit. All analyses were conducted in Prism (version 10.3.0; GraphPad) and JMP (version 17.2.0).

## RESULTS

**Figure 1.**
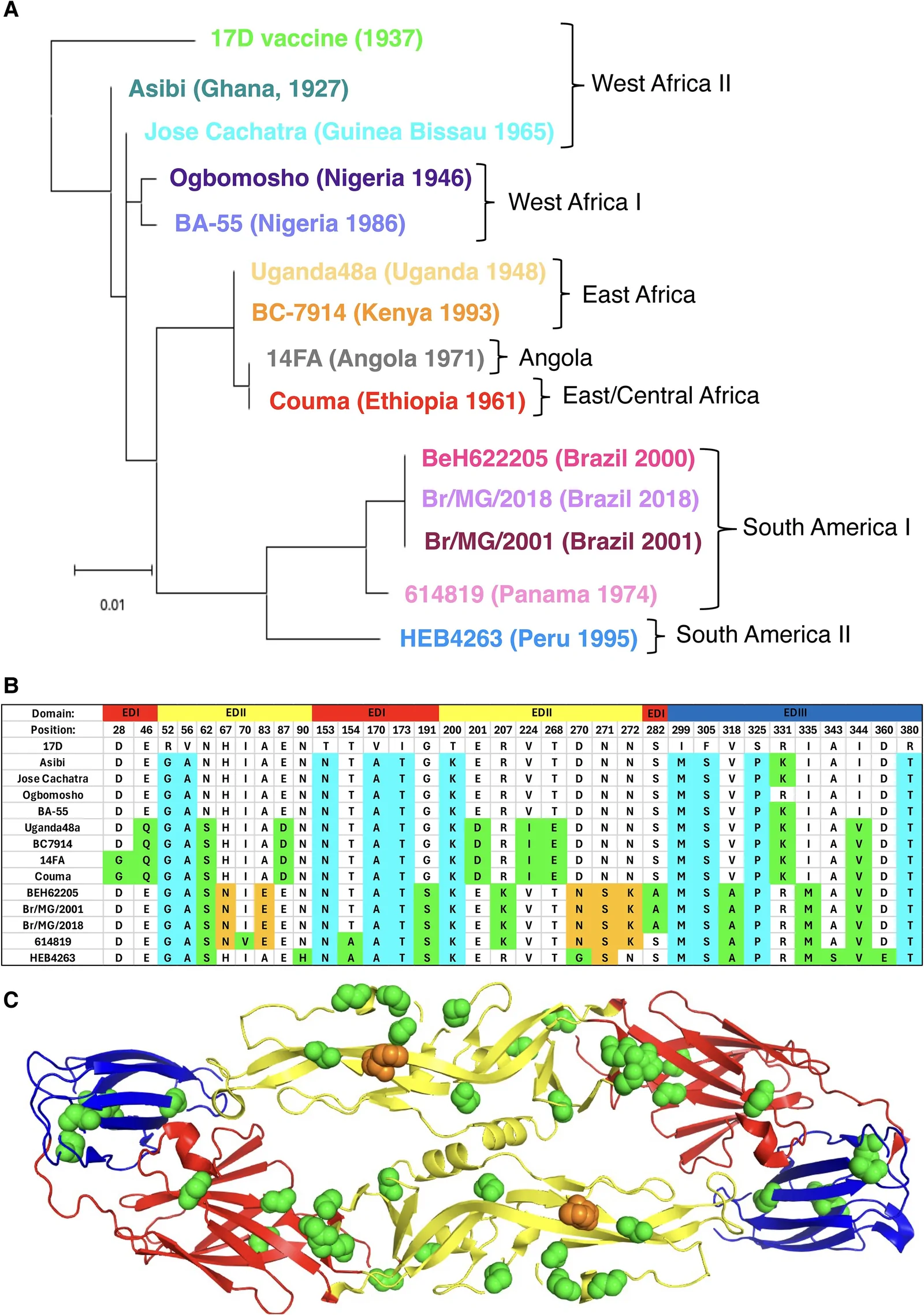
Amino acid changes within the ectodomain of the envelope glycoprotein among wild type yellow fever virus strains. *A*, Phylogenetic tree constructed with the amino acid sequence of the E protein. Virus strain names are colored, and year of isolation is given in parentheses. Genotype names are in black. *B*, Table of amino acid variation within the E glycoprotein ectodomain between 17D and wild type strains, with 33 total amino acid changes: 10 restricted to 17D (cyan) and 23 among wild type viruses (green and orange). Residues implicated in modifying neutralization (Haslwanter et al [11]) restricted to South America genotype I strains are shown in orange. *C*, Ribbon cartoon of yellow fever virus dimer (pdb_00006iw5) with wild type variable sites corresponding to panel *B* shown in green and orange. Image created with the PyMOL Molecular Graphics System (version 3.0; Schrödinger, LLC).

Fourteen viruses were used to characterize immune sera (Figure 1, Supplementary Table 1). The envelope (E) gene of working stocks of each virus was sequenced (Supplementary Table 3), and phylogenetic relatedness was confirmed (Figure 1*A*). We identified 33 amino acid differences across E, with 10 restricted to 17D and 23 among WT viruses (Figure 1*B* and 1*C*). We confirmed the presence of sites implicated by Haslwanter et al [11] to influence 17D NAb potency: H67N, A83E, D270E, and N272K, which are all unique to the SA-I strains, and N271S, which is found in SA-I and SA-II. The 36 study participants (Table 1) had received a single dose of 17D 1 to 11 years prior (median, 6), with age at vaccination ranging from 19 to 69 years (median, 29.5). Vaccinees either were *Orthoflavivirus* naive (n = 18; henceforth, 17D-only vaccinees) or had serology consistent with heterologous *Orthoflavivirus* infection (n = 18; henceforth, heterologous vaccinees), with 10 vaccinated before heterologous infection, 7 after, and 1 with an unknown date of infection (Table 1).

**Table 1.** Study Participants

	No. (%) or Median (Range)
Sex	
Female	28 (77.8)
Male	8 (22.2)
Race and ethnicity	
White	30 (83.3)
Other	5 (13.9)
Unknown	1 (2.3)
Age at vaccination, y	29.5 (19–69)
Years postvaccination	6 (1–11)
17D-only vaccinees	6.5 (2–9)
Heterologous vaccinees	6 (1–11)
*Orthoflavivirus* infection history	
17D-only vaccinees	18 (50.0)
Heterologous vaccinees	18 (50.0)
DENV-1	3 (16.7)
DENV-2	6 (33.3)
DENV-3	1 (5.6)
DENV-4	2 (11.1)
DENV secondary	3 (16.7)
ZIKV	2 (11.1)
DENV and ZIKV	1 (5.6)
Years postinfection (n = 17)^a^	5 (0–26)
Sequence of vaccination/infection (n = 18)^b^	
Vaccination before infection	10
Infection before vaccination	7
Unknown	1

Demographics of study participants, including sex, race and ethnicity, age at vaccination, years postvaccination, and *Orthoflavivirus* infection history.

Abbreviations: DENV, dengue virus; ZIKV, Zika virus.

^a^Date of vaccination was either confirmed by medical history or self-reported.

^b^Date of infection history was self-reported.

**Figure 2.**
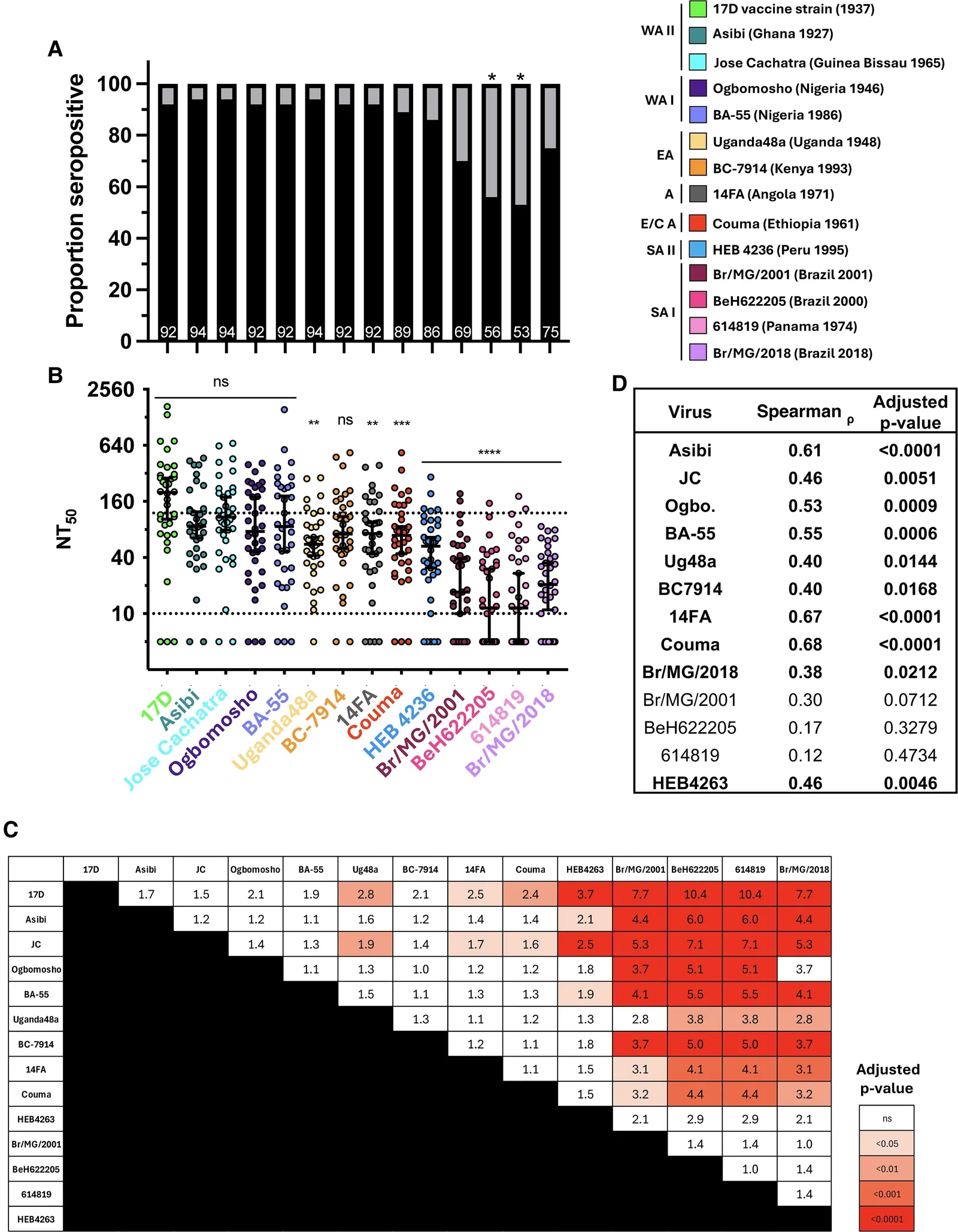
Potency of vaccinee sera against wild type viruses are reduced as compared with 17D. *A*, NT_50_ for all participants against each virus. Each dot represents the geometric mean titer of a single participant’s sera, calculated from biological duplicates. Horizontal bars and whiskers are geometric means with 95% CIs. Lower limit of detection (dotted line) is 1:10 and upper limit of detection is 1:2560. Geometric mean titers <1:10 were given an arbitrary value of 5. Virus strains are color coded according to the key and arranged by genotype. Adjusted *P* values derived from Friedman test with Dunn multiple comparison: **P < .01. ***P < .001. ****P < .0001. ns, not significant. *B*, Proportion of participants who were seropositive (NT_50_ ≥1:10, black) and seronegative (NT_50_ <1:10, gray), with percentage of total (N = 36) shown with white numbers. Chi-square analysis shows a statistically greater proportion of seronegativity for South America genotype I strains Br/MG/2001, BeH622205, and 614819 vs the model prediction. *C*, Spearman rank correlation coefficients and adjusted *P* values between log geometric mean titers of 17D and wild type viruses. Statistically significant values are in bold. *D*, Matrix shows geometric mean titer fold differences among viruses. Boxes are colored by Friedman test with adjusted *P* values per Dunn multiple-comparison test. Abbreviations: A, Angola; EA, East Africa; E/C A, East/Central Africa; NT_50_, 50% neutralizing titer; SA-I, South America I; SA-II, South America II; WA-I, West Africa I; WA-II, West Africa II.

We conducted FRNTs for each of the 14 viruses against each of the 36 immune sera, evaluating NT_50_ and NT_90_ (90% FRNT) titers. We found that NT_50_ titers provided a more informative range of values, as 49% of tested virus-serum combinations had FRNT_90_ titers <1:10 against WT viruses (Supplementary Figure 1). We first determined the proportion of vaccinees who were seropositive against each virus at an NT_50_  *≥*1:10 (Figure 2*A*) and NT_90_ ≥1:10 (Supplementary Figure 1). Over 90% of vaccinees were seropositive by NT_50_ for all strains except SA-I and SA-II, with 86% seropositive against HEB4263, 75% against Br/MG/2018, and 69% against Br/MG/2001, including significantly lower proportions of 56% against BeH622205 and 53% against 614819. By FRNT_90_, the proportions against all SA-I strains were significantly lower at 19% against Br/MG/2018, 28% against Br/MG/2001, 14% against BeH622205, and 17% against 614819 (Supplementary Figure 1).

Friedman test with Dunn multiple comparison found that titers for Uganda48a (adjusted [adj] *P* = .0011), 14FA (adj *P* = .0130), Couma (adj *P* = .0061), HEB4263 (adj *P* < .0001), Br/MG/2001 (adj *P* < .0001), BeH622205 (adj *P* < .0001), 614819 (adj *P* < .0001), and Br/MG/2018 (adj *P* < .0001) were significantly reduced as compared with 17D serum titers (Figure 2*B*). No significant differences were observed between titers within the same genotype. Fold differences for pooled geometric mean titers of each WT virus ranged 1.0 to 10.4 (Figure 2*C*). Notably, FRNT_50_ titers for the SA-I strains—Br/MG/2001, BeH622205, 614819, and Br/MG/2018—were significantly lower than most other viruses, except HEB4263 (Figure 2*B*).

NAbs against 17D are widely accepted as a correlate of protection against WT infection, yet the correlation between 17D and WT YFV titers has not been previously validated. We analyzed the correlation between 17D and each WT virus titer, finding that 17D FRNT_50_ titers were significantly correlated with all WT YFV titers (Spearman ρ, 0.40–0.68; *P* < .0001–.0168) except that SA-I strains Br/MG/2001, BEH622205, and 614819 were not correlated with 17D titers (*P* = .0721–.4734; Figure 2*D*).

We next examined the relationship between E amino acid sequences and NT_50_ titers using Dayhoff distances [20], a weighted measure of amino acid similarity between amino acid sequences, finding a highly significant negative correlation (Pearson *r* = −0.84, *P* = .0002) between WT virus amino acid distance from 17D and WT virus neutralization titers.

**Figure 3.**
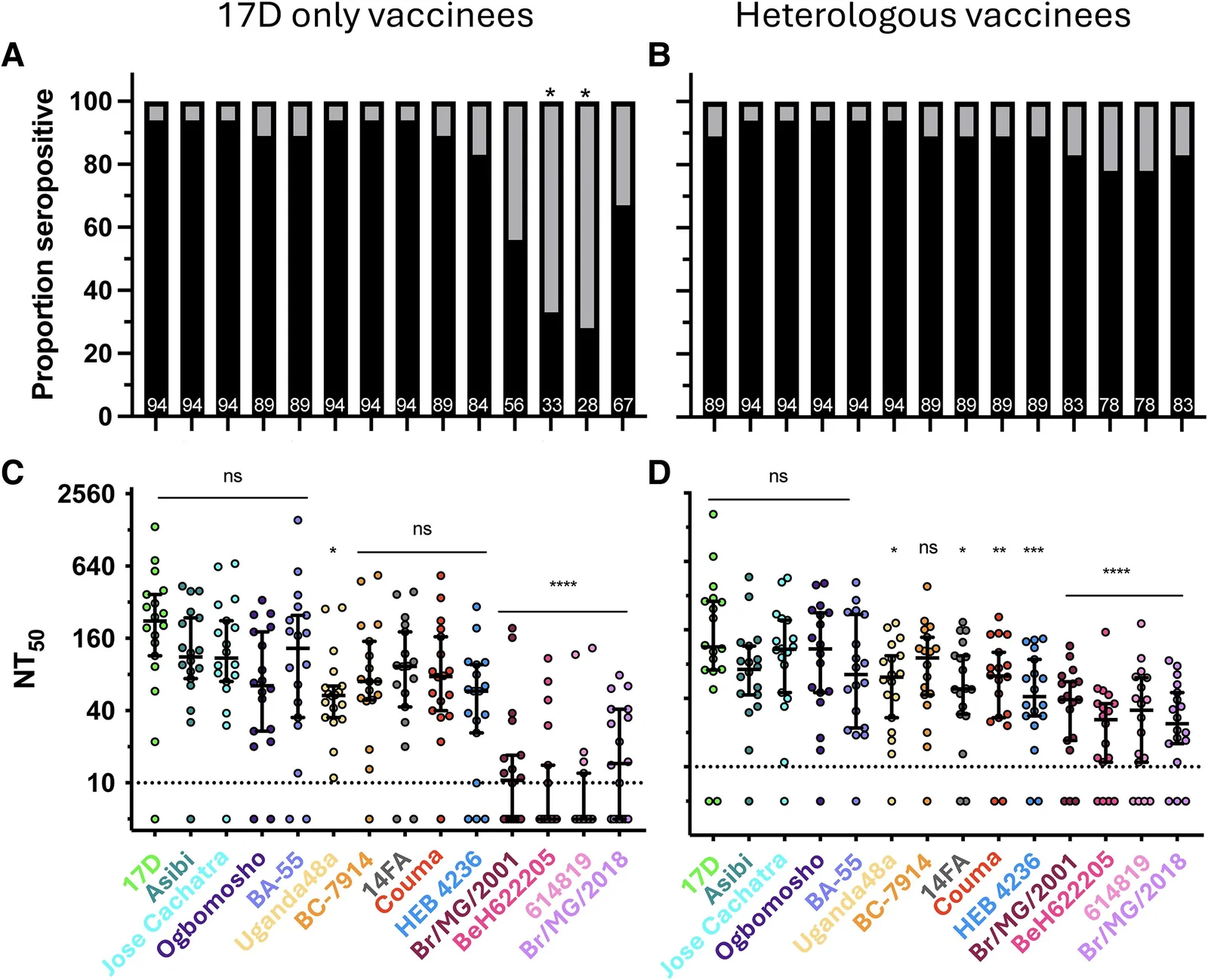
Heterologous vaccinees have increased seropositivity to South America genotype I strains. Virus strains are color coded according to the key in Figure 2 and arranged by genotype. Adjusted *P* values derived from Friedman test with Dunn-multiple comparison: *P < .05. **P < .01. ***P < .001. ****P < .0001. ns, not significant. *A* and *B*, Proportion of seropositivity (NT_50_ ≥1:10, black) and seronegativity (NT_50_ <1:10, gray) among 17D-only and heterologous vaccinees. White numbers indicate percentage of total (n = 18). *P* values derived from analysis of means of proportions: *P < .001. *C* and *D*, NT_50_ against each virus for 17D-only vaccinees and heterologous vaccinees. Each dot represents the geometric mean titer of a single participant’s sera, calculated from biological duplicates. Horizontal bars and whiskers are geometric means with 95% CIs. Lower limit of detection (dotted line) is 1:10 and upper limit of detection is 1:2560. Geometric mean titers <1:10 were given an arbitrary value of 5. Abbreviation: NT_50_, 50% neutralizing titer.

The impact of heterologous *Orthoflavivirus* immunity on 17D-elicited immunity against WT YFV has not, to our knowledge, been previously examined. We first stratified titers by *Orthoflavivirus* infection history and compared serostatus between 17D-only and heterologous vaccinees. We observed significantly reduced proportions of seropositivity against WT among 17D-only vaccinees, with 33% against BeH622205 (*P* < .001) and 28% against 614819 (*P* < .001; Figure 3*A*). Br/MG/2001 (56%) and Br/MG/2018 (67%) trended toward a reduced proportion of seropositive vaccinees but were not significant (*P* > .05), while by FRNT_90_, seropositivity was significantly reduced against all 4 SA-I viruses (Supplementary Figure 1). In contrast, heterologous vaccinees did not have significantly reduced seropositivity against SA-I viruses, with 83% seropositive against Br/MG/2001, 78% against BeH622205, 78% against 614819, and 83% against Br/MG/2018 (Figure 3*D*), with ≥89% seropositive for all other viruses (Figure 3*A* and 3*B*), although by FRNT_90_, seropositivity was significantly lower for BeH622205 (Supplementary Figure 1).

We next compared NT_50_ geometric mean titers among 17D-only vaccinees, and titers against Uganda48a (adj *P* = .0214) and HEB4263 (adj *P* = .0006), as well as SA-I strains Br/MG/2001 (*P* < .0001), BeH622205 (adj *P* < .0001), 614819 (adj *P* < .0001), and Br/MG/2018 (adj *P* < .0001), were significantly reduced vs 17D (Figure 3*A*). Among heterologous vaccinees, titers against Uganda48a (adj *P* = .0300), 14FA (adj *P* = .0200), Couma (adj *P* = .0086), HEB4236 (adj *P* = .0008), Br/MG/2001 (adj *P* < .0001), BeH622205 (adj *P* < .0001), 614819 (adj *P* < .0001), and Br/MG/2018 (adj *P* < .0001) were significantly reduced when compared with 17D (Figure 3*C*).

We calculated fold differences between pooled geometric mean titers for every virus pair (Supplementary Figure 2), and more significant differences were observed between 17D-only vaccinees (34/91 pairs) and heterologous vaccinees (20/91 pairs), with 17D-only fold differences ranging 1.0 to 20.7 as compared with 1.0 to 6.0 for heterologous vaccinees.

Last, we conducted pairwise comparisons for 17D-only and heterologous vaccinee titers for each virus (Supplementary Figure 3), finding that titers of heterologous vaccinees were significantly higher than 17D-only vaccinees against all SA-I strains (*P* < .0001) and individually against Br/MG/2001 (*P* = .0109), BeH622205 (*P* = .0271), and 614819 (*P* = .0051). Titers against Br/MG/2018 trended higher for heterologous vaccinees, although this difference was not significant (*P* = .2494). We assessed whether the sequence of vaccination/infection affected virus-specific NT_50_ titers for heterologous vaccinees, finding a trend toward vaccination after infection leading to lower 17D NT_50_ titers (*P* = .0514) but no significant associations for the WT viruses, with *P* values ranging from .158 (Ogbomosho) to .9594 (BR/MG 2018). No association was found between NT_50_ or NT_90_ serostatus and vaccination/infection sequence.

**Figure 4.**
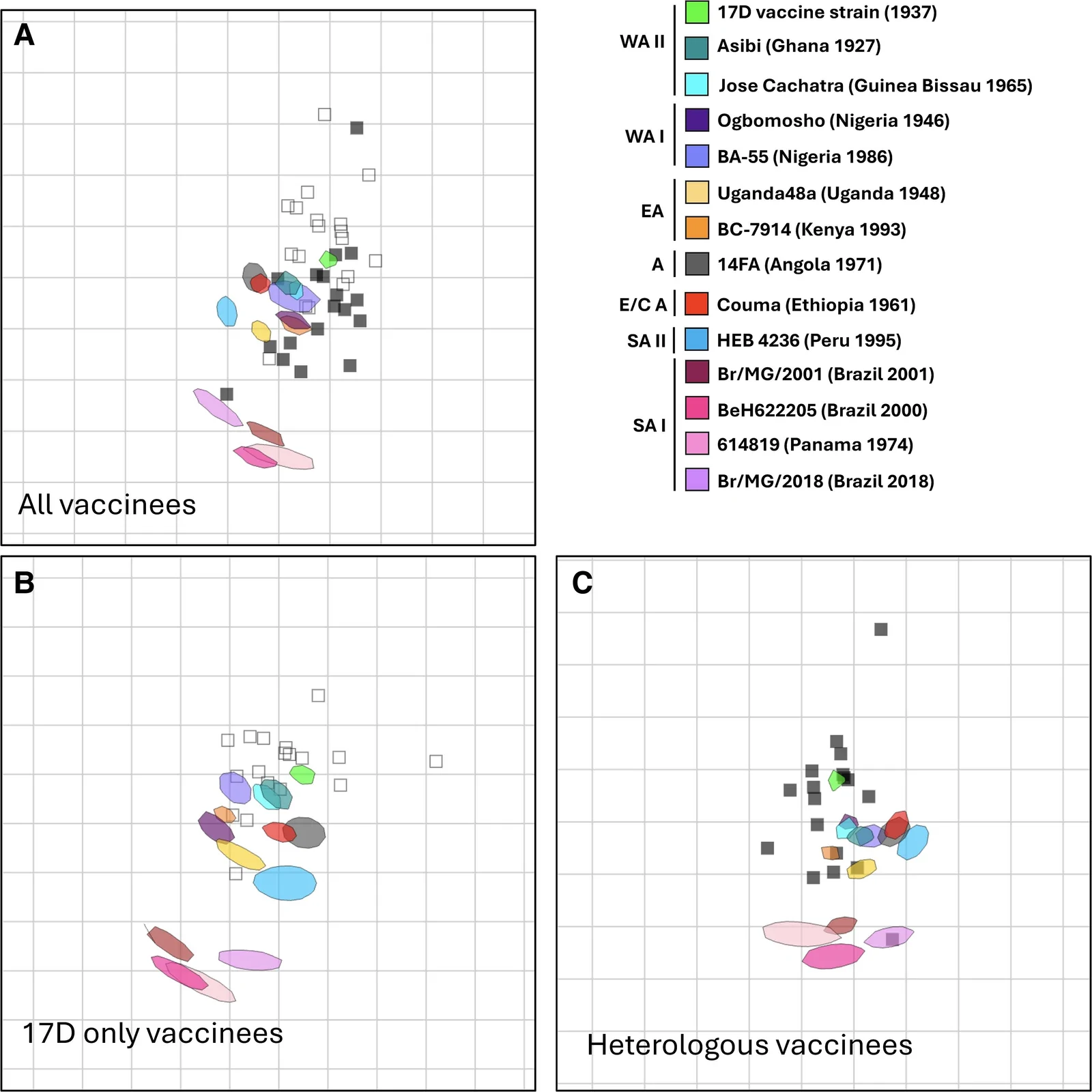
Antigenic cartography of 17D and wild type yellow fever virus strains, using 17D-immune sera. *A–C*, Antigenic cartography maps generated with all 17D vaccinee sera (N = 36), as well as 17D only and heterologous. Open squares, 17D only; filled squares, heterologous. Maps were generated via the Racmacs package in R (version 4.3.1) and RStudio (version 2023.06.1 + 524). A single gridline-defined box represents 2-fold serum dilution of a neutralization titer. Abbreviations: A, Angola; EA, East Africa; E/C A, East/Central Africa; SA-I, South America I; SA-II, South America II; WA-I, West Africa I; WA-II, West Africa II.

Antigenic cartography is used to visualize the antigenic relationship among virus strains using NT titers [17–19, 21, 22]. Antigenic maps place individual viruses and immune sera in a multidimensional space in which positions are optimized such that the straight-line distances for each virus-serum pair best matches pairwise neutralization titers in a 2 × 2 matrix [18, 23]. As expected, SA-I strain viruses were most antigenically distant from 17D, forming a distinct cluster (Figure 4*A*), with distances of 3.16 to 3.69 antigenic units for SA-I as compared with 1.00 to 1.75 for non-SA strains (Supplementary Table 4). We next stratified maps by *Orthoflavivirus* infection history, finding that the antigenic distances between sera and SA-I strains increased for 17D-only vaccinee titers (3.95–4.64; Figure 6*B*) and decreased for heterologous vaccinee titers (2.26–2.88; Figure 6*C*).

**Figure 5.**
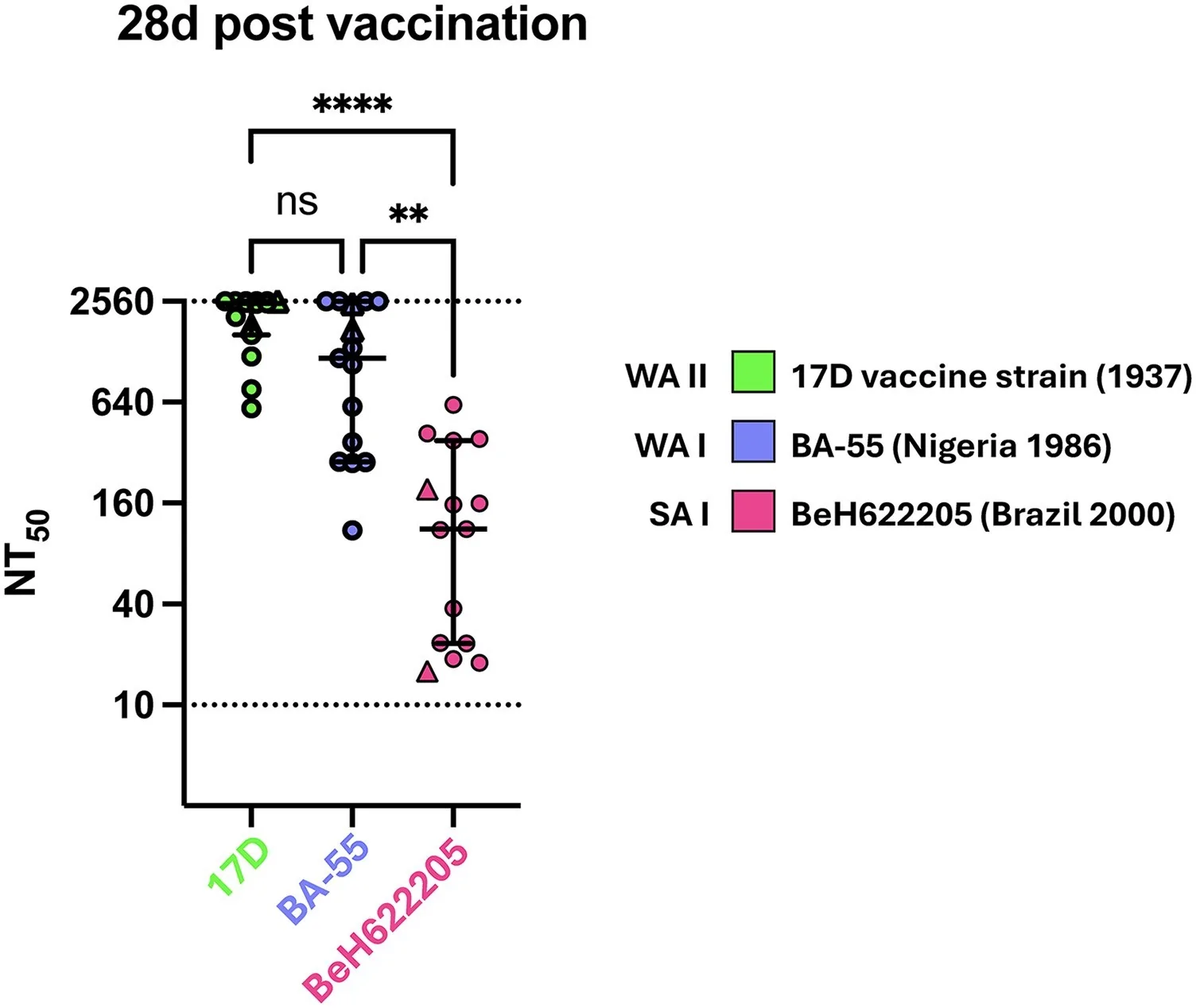
Reduced potency against the South America genotype I is observed 28 days postvaccination. NT_50_ geometric mean titers (GMTs) of vaccinee sera 28 days postvaccination against representative wild type viruses. Each dot represents the GMT of a single participant’s sera, calculated from biological duplicates. Lower limit of detection (dotted line) is 1:10 and upper limit of detection is 1:2560. GMTs <1:10 were given an arbitrary value of 5. Adjusted *P* values derived from Friedman test with Dunn multiple comparison: **P < .01. ****P < .0001. ns, not significant. Triangles indicate participants with low-level DENV2 seropositivity. Abbreviations: NT_50_, 50% neutralizing titer; SA-I, South America I; WA-I, West Africa I; WA-II, West Africa II.

To assess the potency and breadth of 17D-immune sera immediately following vaccination, we performed FRNTs on 15 adult vaccinee sera at 28 days postvaccination against a subset of our WT virus panel (Figure 5). Study participants were aged 23 to 49 years (median, 30) at vaccination and 43% were female. Two had low-level FRNT_50_ titers against DENV2 (1:26 and 1:28) but were otherwise DENV seronegative. Postvaccination, all vaccinees were seropositive against all viruses, but titers against SA-I strain BeH622205 were significantly reduced when compared with 17D (adj *P* < .0001) and BA-55 (adj *P* = .0042; Figure 5), and 6 sera had an FRNT_50_ <1:40 against BeH622205 at 28 days postvaccination.

**Figure 6.**
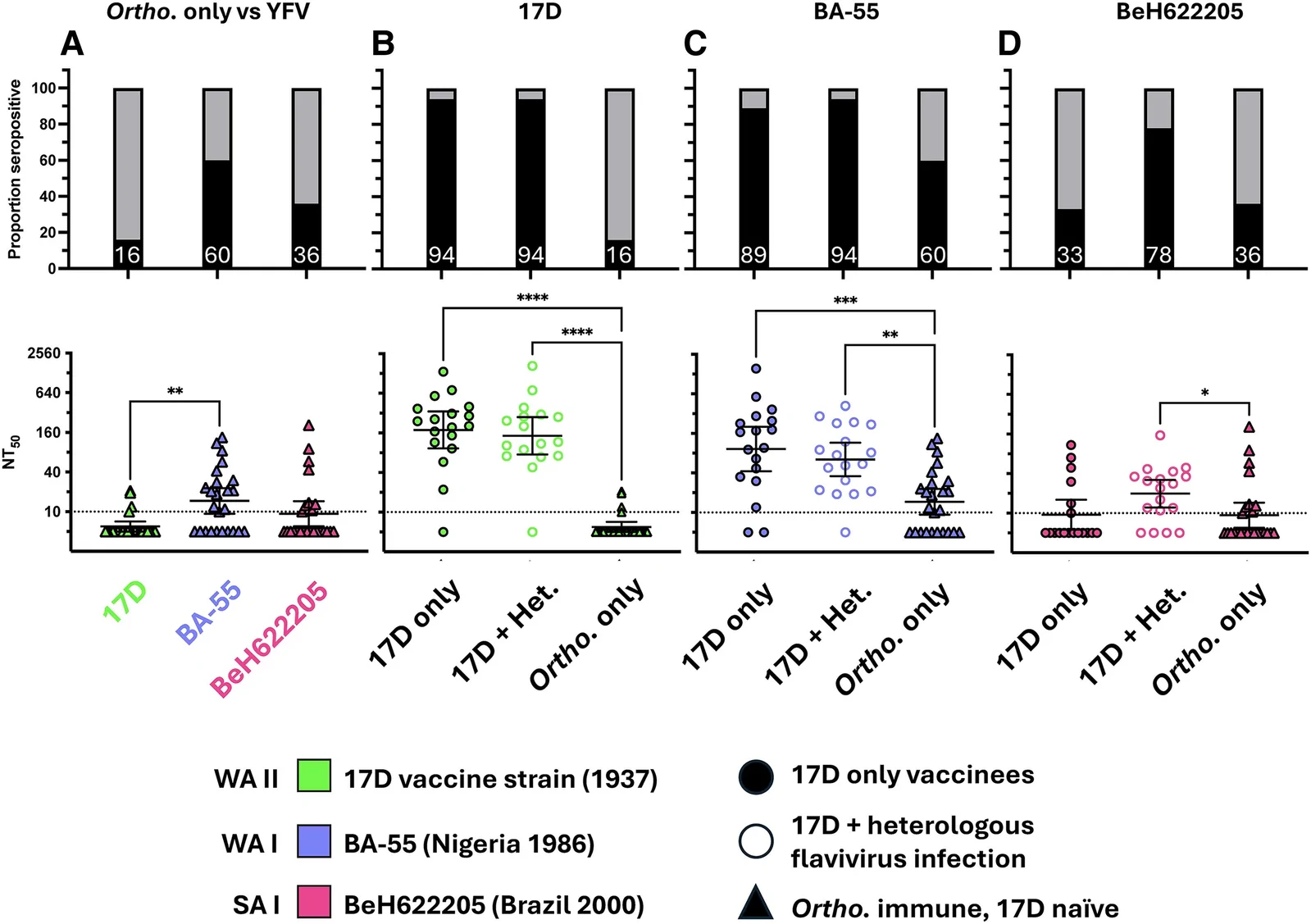
YFV cross-reactivity of individuals who were *Orthoflavivirus* immune and unvaccinated. Top panels show the proportion of participants who were seropositive (NT_50_ ≥1:10, black) and seronegative (NT_50_ <1:10, gray), with percentage of total (n = 25) shown with white numbers. Bottom panels indicate the NT_50_ against viruses. Each dot represents the GMT of a single participant’s sera, calculated from biological duplicates. Horizontal bars and whiskers are geometric means with 95% CIs. Lower limit of detection (dotted line) is 1:10 and upper limit of detection is 1:2560. Titers <1:10 were given an arbitrary value of 5. Virus strains are color coded according to the key. Adjusted *P* values derived from Friedman test with Dunn multiple comparison: *P < .05. **P < .01. ***P < .001. ****P < .0001. *A–D*, Abbreviations: DENV-immune sera against viruses, followed by 17D-only vaccinees (filled circles), heterologous vaccines (open circles), and DENV-immune sera (triangles) against 17D, BA-55, and BeH622050. DENV, dengue virus; NT_50_, 50% neutralizing titer; SA-I, South America I; WA-I, West Africa I; WA-II, West Africa II; YFV, yellow fever virus.

We next assessed the cross-reactivity of 25 nonvaccinated *Orthoflavivirus*-immune sera (Supplementary Table 5) against WT YFV, a comparison that has not been previously made (Figure 6). Of 25 sera, 4 (16%) were seropositive against 17D, with titers ranging from 1:12 to 1:21 (Figure 6*A* and 6*B*). Cross-reactivity was greatest against the West Africa I strain BA-55, with 15 of 25 (60%) seropositive with a titer range of 1:10 to 1:135, significantly lower than 17D-only vaccinees (adj *P* = .0001) and heterologous vaccinees (adj *P* = .0021; Figure 6*A* and 6*C*). Cross-reactivity against SA-I strain BeH622205 was limited, with 9 of 25 (36%) seropositive with a titer range of 1:12 to 1:207 (Figure 6*A* and 6*D*). There was no significant difference in FRNT_50_ titers between DENV and 17D-only immunity (adj *P* = .9999), but heterologous vaccinee titers were significantly higher than DENV immunity (adj *P* = .0220; Figure 6*D*).

## DISCUSSION

Most studies evaluating 17D-elicited NAb responses have used vaccine strain 17D and occasionally the parental Asibi strain as test viruses [3, 24, 25]. This constraint is logistical: WT YFV is handled at biosafety level 3, and assembling a test panel of viruses is nontrivial. 17D is a convenient, fully homotypic, biosafety level 2 test virus. To our knowledge, ours is the most comprehensive characterization of the potency and breadth of 17D-vaccinee sera with diverse vaccination and host immune backgrounds, with the largest differences observed against SA-I strains, consistent with Haslwanter et al [11] and Goncalves et al [12]: the former reported a ∼7- to 8-fold reduction in FRNT_50_ between 17DD and SA-I strain ES-504, and the latter reported ∼2- to 3-fold reduction between 17DD and SA-I strain Hu-BR2018, which are similar to the fold reductions in our heterologous vaccinees (4.7–7.1). Using our library of authentic viruses, including 4 SA-I strains, we confirmed all 5 amino acid substitutions identified by Haslwanter et al in SA-I strains Br/MG/2001, BeH622205, BR/MG/2018, and 614819. Notably, 614819 was isolated in Panama in 1974, demonstrating that these mutations arose at least 42 years before the recent outbreak in Brazil. We identified the N271S mutation in our representative SA-I strain HEB4263, which trended toward decreased titers and fewer seropositive vaccinees as compared with 17D and other African WT viruses, supporting the previous finding that N271S alone impedes neutralization by 17D-immune sera [11].

We also identified a key modulating host factor—heterologous *Orthoflavivirus* infection—that significantly increased the potency and breath of 17D-immune sera against SA-I strains and the proportion of vaccinees who were seropositive against SA-I strains. These data suggest that prior or subsequent immunity elicited by heterologous *Orthoflavivirus* infection may effectively “boost” immunity, a finding with implications for 17D vaccination strategies between endemic and nonendemic populations. Interestingly, Haslwanter et al found 88% (21/24) of Brazilian vaccinees to be seropositive against the SA-I strain ES-504 [26], similar to our heterologous vaccinees against SA-I strains (72%–83%). DENV is endemic in Brazil, which experienced an unprecedented ZIKV outbreak in 2015–2016, making it possible that their cohort included heterologous vaccinees, something that the authors did not test. Additionally, Shinde et al [27] reported that DENV or ZIKV immunity suppressed YFV viremia in a WT YFV challenge model in cynomolgus macaques, and the authors posited that DENV and ZIKV immunity may limit YFV transmission in high-seroprevalence settings. While we found evidence of cross-neutralization of WT YFV by DENV-immune sera, the extent and magnitude of the cross-reactivity alone does not explain the greater potency and breadth of heterologous vaccinee sera against SA-I YFV strains.

It is possible that T-cell–mediated immunity may add a layer of protection not measured by our study. 17D elicits robust T-cell responses postvaccination [28]. While a recent study [29] reported that T cells were protective against viremia in a 17D → chimeric 17D/Japanese encephalitis virus human challenge model at 28 days postvaccination, earlier studies using 17D/Japanese encephalitis virus [30] or 17D/DENV2 [31] chimeric vaccines with a >10-month interval between vaccination and booster found no evidence of T-cell–mediated protection against viremia, suggesting that T-cell immunity wanes and likely plays a subordinate role in long-term protection [32].

Our antigenic maps revealed that SA-I strains form a distinct antigenic cluster by 17D-only and heterologous neutralization titers. Beyond defining the antigenic landscape of YFV, continued application of antigenic cartography could enhance surveillance of circulating YFV, facilitate risk prediction, and potentially inform future vaccine design strategies.

Our work does not establish the molecular basis for increased potency of heterologous immune sera. However, the N67 mutation in SA-I WT YFV is otherwise unique to and highly conserved across DENV serotypes. For DENV, N67 contributes to a glycosylation site that interacts with host cell receptor DC-SIGN (dendritic cell–specific ICAM3-grabbing nonintegrin) [33], which is required for DENV infectivity and pathogenesis [34]. Yet, the YFV N67 mutation does not form the canonical N-linked glycosylation motif NxS/T [35], instead forming an NVK sequence, but it is striking that 36% of our *Orthoflavivirus-*only sera included antibodies that neutralized BEH622205. We hypothesize that DENV infection results in the generation of memory B cells (MBCs) capable of recognizing N67 epitopes that are cross-reactive with SA-I strains. Similarly, we hypothesize that vaccination with 17D may elicit MBCs that are cross-reactive against DENV strains. Under these hypotheses, MBC recall responses to DENV infection or 17D vaccination provide secondary antigen recognition and affinity maturation, resulting in differentiation of MBCs into plasma cells that secrete antibodies with increased cross-reactivity to N67 epitopes.

Our study's strengths include the use of a diverse and authentic WT YFV panel, a balanced distribution of years postvaccination, and a distribution of ages at vaccination that is particularly representative of traveler populations worldwide.

Our study's limitations include a relatively small cohort, lacking children and individuals vaccinated over the age of 69 years and overrepresented by White and female participants. Moreover, some statistical analyses should be interpreted cautiously, particularly for our smaller heterologous and 17D-only vaccinee subsets. Our data also suggest the potential for an increased risk of breakthrough infection with SA-I strains, which calls for further evaluation of this risk. 17D breakthrough infections are not systematically reported [36], and the extent to which breakthrough infections have or have not contributed to cases in South America in the past decade is not known. With recent outbreaks and ongoing sylvatic transmission that maintains viral reservoirs within which viral evolution continues, YFV continues to pose a threat to public and global health.

## Supplementary Material

jiag077_Supplementary_Data

## Supplementary Data

Supplementary materials are available at *The Journal of Infectious Diseases* online (http://jid.oxfordjournals.org/). Supplementary materials consist of data provided by the author that are published to benefit the reader. The posted materials are not copyedited. The contents of all supplementary data are the sole responsibility of the authors. Questions or messages regarding errors should be addressed to the author.

## Notes

***Acknowledgments.*** We acknowledge Brian Booty and Peter Sullivan from OHSU and Tara Beatty, Evelin Coto, Lantoria Davis, Stephen Fortmann, Tarika Holness, Kristin Muessig, Elizabeth Shuster, and Mica Werner from the Kaiser Permanente Center for Health Research for their contributions to coordination of the cohorts in this study. We also acknowledge the World Reference Center for Emerging Viruses and Arboviruses for the provision of virus strains Couma, Uganda48a, Ogbomosho, Jose Cachatra, HEB4236, and Asibi. Additionally, we acknowledge the Centers for Disease Control and Prevention’s Arbovirus Reference Collection for the provision of virus strains Br/MG/2001, BeH622205, BC-7914, BA-55, 614819, and 14FA. Thank you to Betania Drumond, PhD, Universidade Federal de Minas Gerais, and to Alec Hirsch, PhD, Vaccine and Gene Therapy Institute, OHSU, for generously providing the 321/Br/MG/2018 virus and the 4G2 antibody, respectively.

***Data availability.*** Data are available on request.

***Financial support.*** This work was supported by the National Institutes of Health R01AI145835-01A1 to W.B.M.; R01AI153434-01A1 to W.B.M.; R21AI135537 to W.B.M; the Sunlin and Priscilla Chou Foundation (to W.B.M.); Oregon Clinical & Translational Research Institute (UL1TR002369); and Takeda Vaccines (IISR2016-10-101586 to W.B.M.).
