# Supplementary material for: New Insights Into an Old Vaccine: Potency and Breadth of Neutralizing Antibodies Elicited by Yellow Fever Vaccine 17D Are Boosted by Heterologous Orthoflavivirus Infection
Source: J Infect Dis. 2026 Feb 11;234(2):e232–42. doi: 10.1093/infdis/jiag077 (PMC13431080; doi:10.1093/infdis/jiag077)
Supplement: Supplementary file 1 [file jiag077_supplementary_data.docx]

Supplemental Material

| **Strain** | **Date of isolation** | **Location of Isolation** | **Genotype** | **Host source** | **Isolate history** | **Contributor history** | **Passage history (passage #)**  ***** |
| --- | --- | --- | --- | --- | --- | --- | --- |
| Br/MG/2001 | Mar-2001 | Bom Despacho, Minas Gerais, **Brazil** | South America I | *Homo sapiens* | Male, 39 yo. Isolated from serum, during sylvatic outbreak. Patient died 3/7/2001 | FIOCRUZ  ↓  Barbara Johnson at ADB RRL  ↓  ARC (lot: TC01532)  ↓  OHSU | Human  ↓  C636 (p1)  ↓  Vero (p2)  ↓  Vero (p3)  ↓  Vero (p4) |
| BeH622205 | 3-Mar-2000 | Goiás, **Brazil** | South America I | *Homo sapiens* | No further isolate history provided | Pedro Vasconcelos, Belem Virus Lab, Evandro Chagas Institute  ↓  Rober Tesh, WRCEVA, 2013  ↓  ARC (lot: TC00892 WSV, 2013)  ↓  OHSU | Human (p0)  ↓  SM (p1)  ↓  C636 (2; p2-3)  ↓  Vero (p4)  ↓  Vero (p5) |
| BC-7914 | 1993 | Baring, **Kenya** | East Africa | *Homo sapiens* | No further isolate history provided | Bruce C. Cropp, CDC (1998)  ↓  ARC (lot: TC00884 WSV, 2012)  ↓  OHSU | Human  ↓  P?  ↓  SM  ↓  C636  ↓  Vero  ↓  Vero  ↓  Vero |
| BA-55 | 1986 | **Nigeria** | West Africa I | *Homo sapiens* | No further isolate history provided | Bruce C. Cropp, CDC (1998)  ↓  ARC  ↓  OHSU | Human  ↓  P?  ↓  SM (2)  ↓  Vero E6  ↓  Vero  ↓  Vero |
| 614819 | 1974 | **Panama** | South America I | *Homo sapiens* | No further isolate history provided | MARU (WRAIR)  ↓  Thomas Monath, CDC (1976)  ↓  ARC (lot: MARU1974WSV)  ↓  OHSU | Human (p0)  ↓  Night monkey (p1)  ↓  Vero (p3)  ↓  Vero (p4) |
| 14FA/ Angola71 | 1971 | **Angola** | Angola | *Homo sapiens* | ARC | YARU, Yale University  ↓  ARC  ↓  OHSU | SM (p1-7)  ↓  Mosquito (p8)  ↓  C636 (p9)  ↓  Vero (p10)  ↓  Vero (p11)  ↓  Vero (p12) |
| Asibi | 1927 | Kpeve Village, **Ghana** | West Africa II | *Homo sapiens* | Male, 28 yo. Patient survived. | YARU, Yale University  ↓  WRCEVA  ↓  OHSU | Human  ↓  Monkey (6)  ↓  C636 (3)  ↓  Vero  ↓  Vero |
| Couma | Jun-1961 | **Ethiopia** | East/Central Africa | *Homo sapiens* | No further isolate history provided | Institute Pasteur Paris, France  ↓  YARU  ↓  WRCEVA  ↓  OHSU | Human  ↓  SM (46)  ↓  Mosquito (2)  ↓  Vero  ↓  Vero |
| HEB 4236 | 5-Mar-1995 | **Peru** | South America II | *Homo sapiens* | No further isolate history provided | D. Watts, NAMRID, Peru  ↓  WRCEVA  ↓  OHSU | Human  ↓  C636  ↓  Vero  ↓  Vero |
| Ogbomosho | 1946 | Ogbomosho, **Nigeria** | West Africa I | *Homo sapiens* | No further isolate history provided | YARU  ↓  WRCEVA  ↓  OHSU | Human  ↓  P?  ↓  C636 (2)  ↓  Vero  ↓  Vero |
| Uganda48a/ MR896 | Jul-1948 | **Uganda** | East Africa | *Aedes* spp. | No further isolate history provided | Institute Pasteur Bangai, Central African Republic  ↓  YARU  ↓  WRCEVA  ↓  OHSU | Mosquito  ↓  C636  ↓  Vero  ↓  Vero |
| Jose Cachatra | 23-Aug-1965 | **Guinea Bissau** | West Africa II | *Homo sapiens* | No further isolate history provided | YARU  ↓  WRCEVA  ↓  OHSU | Human  ↓  SM (6)  ↓  Mosquito  ↓  Vero  ↓  Vero |
| 321_BR_MG_2018  “Br/MG/2018” | 2018 | Minas Gerais, **Brazil** | South America I | *Homo sapiens* | Female, 71 yo. Patient Survived.  No further isolate history provided | Instituto de Ciências Biológicas, Universidade Federal de Minas Gerais, Brazil  ↓  OHSU | Human  ↓  Vero (2)  ↓  C636 (4)  ↓  Vero  ↓  Vero |

**Supplemental Table 1. Virus origin and passage histories.**

*Indicates working stocks

Abbreviations

ADB RRL = ADB Reference and Reagent Lab

ARC = Arbovirus Reference Collection (CDC)

CDC = Centers for Disease Control and Prevention

FIOCRUZ = Oswaldo Cruz Foundation

MARU = Middle America Research Unit

NAMRID = Naval Medical Research Institute Detachment

P? = unknown passage history

WRAIR = Walter Reed Army Institute of Research

WRCEVA = World Reference Center for Emerging Viruses and Arthropod-borne viruses

YARU = Yale Arbovirus Research Unit

SM = suckling mouse

| **Primer** | **Sequence 5’-3’** |
| --- | --- |
| **857R_YFVx** | AGCCATCTCTCAATCTTYTGS |
| **996F_YFV** | YGACAGRGAYTTCATTGAGGG |
| **1300F_YFV** | GGGAAAGGRAGCATTGTGG |
| **1520R_YFV** | CAYTCCAGTGTRGCTTTYCC |
| **2144F_YFV** | ACAAAGARGGAAGYTCAATAGG |
| **2340R_YFV** | GACCTTTGTTATCCAGYTCAAGC |
| **2843F_YFV** | ATGGAAGCTTCATCATAGAYGG |
| **3010R_YFV** | CACCCARGATRGATCCATCR |
| **YFVAmp1A** | ACCTTCAAGAGGTGTTCAAGG |
| **YFVAmp2A** | CTCCTCCATTGTTCATCTCATG |
| **YFVSeq2A_R** | CTATCATCATGCTCACCAAMCC |
| **1805F_Couma** | GGTGGTCATGTCTCTT |
| **2466R_Couma** | GCACATCCTTGGTCTGCC |
| **3011R_Couma** | TGCACCTAGTATAGCC |

**Supplemental Table 2: YFV Sequencing primers**

| **Virus strain** | **Accession number** |
| --- | --- |
| 321_BR_MG_2018 | PX378890 |
| 14FA | PQ241465 |
| BR_MG_2001 | PQ241466 |
| HEB4236 | PQ241467 |
| Ogbomosho | PQ241468 |
| 614819 | PQ241469 |
| Asibi | PQ241470 |
| BA-55 | PQ241471 |
| BC-7914 | PQ241472 |
| BeH622205 | PQ241473 |
| Couma | PQ241474 |
| Jose_Cachatra | PQ241475 |
| Uganda48A | PQ241476 |
| 17D_vaccine | PQ241477 |

**Supplemental Table 3. Virus sequence accession numbers.**

**Supplemental Figure 1. FRNT_90_ Serostatus of study participants. A)** By all participants, dotted line is at 50%. **B)** By 17D only vaccinees, and **C)** by Heterologous immune vaccinees. * indicate viruses with significantly different proportions seropositive by analysis of means for proportions (APOM), P=0.05, adjusted for multiple comparisons.

*

*

*

*

*

*

*

*

*

*

**Supplemental Figure 2. Heterologous vaccinees have increased potency against SA-I strains compared to 17D-only vaccinees.** Individual plots show GMTs of 17D only- (open circles, right) and heterologous vaccinees (filled circles, left) against a single virus, with virus strain and genotype indicated above each frame. Horizontal lines indicate medians, and p-values (Mann-Whitney U) are indicated for each plot. Statistically significant p-values are in bold and color. SA I Strains is a compilation of all sera SA 1 NT test titers.

**Supplemental Figure 3.** Matrix showing fold differences between NT_50_ GMTs between viruses. By **A)** 17D only and **B)** Heterologous vaccinees. Individual frames show fold differences and are colored by adjusted p-values derived from Friedman test with Dunn’s multiple comparisons test**.**

| **Virus strain** | **All vaccinees** | **17D only** | **Heterologous** | **Map distance differences (17D only versus heterologous)** | **Fold-antigenic difference (17D only versus heterologous)** |
| --- | --- | --- | --- | --- | --- |
| 17D vaccine | 0.96 | 0.85 | 1.1 | 0.11 | 1.1 |
| Asibi | 1.13 | 1.30 | 1.2 | 0.17 | 1.2 |
| Jose Cachatra | 1.11 | 1.12 | 1.0 | 0.02 | 1.0 |
| Ogbomosho | 1.86 | 1.09 | 1.7 | 0.76 | 1.7 |
| BA-55 | 1.31 | 1.35 | 1.0 | 0.04 | 1.0 |
| Uganda48a | 1.95 | 1.50 | 1.3 | 0.44 | 1.3 |
| BC-7914 | 1.55 | 1.16 | 1.3 | 0.39 | 1.3 |
| 14FA | 1.80 | 1.66 | 1.1 | 0.14 | 1.1 |
| Couma | 1.66 | 1.66 | 1.0 | 0.00 | 1.0 |
| HEB4263 | 2.47 | 1.97 | 1.3 | 0.50 | 1.3 |
| Br/MG/2001 | 4.17 | 2.26 | 1.8 | 1.91 | 1.8 |
| BEH622205 | 4.61 | 2.88 | 1.6 | 1.73 | 1.6 |
| 614819 | 4.65 | 2.60 | 1.8 | 2.05 | 1.8 |
| Br/MG/2018 | 3.95 | 2.33 | 1.7 | 1.62 | 1.7 |

**Supplemental Table 4: Antigenic distances between virus strains and sera.** Table of median antigenic distances between sera and viruses, extracted from antigenic cartography models constructed using NT_50_ of total-, 17D-only-, and heterologous vaccinee sera (Figure 4A, B, and C). Values represent antigenic units corresponding to a single box within the antigenic map, where each box represents a two-fold serum dilution of a neutralization titer.

| ID | DV1 | DV2 | DV3 | DV4 | ZIKV | YFV 17D | YFV BA-55 | YFV BEH | Years post (last suspected infection) |
| --- | --- | --- | --- | --- | --- | --- | --- | --- | --- |
| 1 | <20 | 25 | <20 | 149 | <20 | <10 | 28 | 10 | 22.5 |
| 2 | 101 | <20 | <20 | <20 | <20 | <10 | <10 | <10 | 39.7 |
| 3 | <20 | <20 | 64 | <20 | <20 | <10 | 21 | <10 | 15.7 |
| 4 | 58 | 103 | 99 | 30 | <20 | <10 | <10 | <10 | 1.0 |
| 5 | <20 | 99 | 1501 | <20 | <20 | <10 | <10 | <10 | 14.9 |
| 6 | <20 | <20 | <20 | 22 | <20 | 21 | <10 | <10 | 3.2 |
| 7 | <20 | <20 | 741 | <20 | <20 | <10 | 10 | <10 | 8.1 |
| 8 | <20 | 1705 | <20 | <20 | <20 | <10 | 11 | <10 | 4.5 |
| 9 | 312 | <20 | <20 | 25 | 24 | <10 | 42 | 10 | 7.5 |
| 10 | 217 | 57 | 148 | <20 | <20 | 12 | 135 | 90 | 3.8 |
| 11 | 55 | 262 | 117 | 30 | 765 | 19 | 85 | 58 | 73.0 |
| 12 | 188 | 77 | 34 | <20 | <20 | <10 | 21 | <10 | 7.7 |
| 13 | 236 | 29 | <20 | <20 | <20 | <10 | <10 | <10 | 7.7 |
| 14 | <20 | 35 | 44 | 48 | <20 | <10 | 12 | <10 | 1.9 |
| 15 | <20 | 103 | <20 | <20 | <20 | <10 | <10 | <10 | 3.0 |
| 16 | <20 | <20 | <20 | 49 | <20 | <10 | <10 | <10 | 2.9 |
| 17 | 556 | 25 | 109 | 76 | 722 | <10 | 110 | 207 | 1.7 |
| 18 | <20 | 279 | <20 | <20 | <20 | <10 | <10 | <10 | 2.8 |
| 19 | 578 | 87 | 353 | 114 | 47 | <10 | 57 | 44 | 3.3 |
| 20 | <20 | 76 | 708 | <20 | <20 | 10 | 30 | 12 | 11.2 |
| 21 | <20 | <20 | <20 | 23 | <20 | <10 | <10 | <10 | 2.3 |
| 22 | 366 | 61 | 125 | 208 | 21 | <10 | 23 | 14 | 25.5 |
| 23 | <20 | <20 | 2703 | <20 | <20 | <10 | 18 | <10 | 3.7 |
| 24 | 226 | <20 | 30 | <20 | <20 | <10 | <10 | <10 | 30.1 |
| 25 | 9336 | 895 | 567 | 65 | <20 | <10 | 20 | 13 | 18.3 |

**Supplemental Table 5. Neutralization titers for *Orthoflavivirus* immune 17D naïve samples.** FRNT_50_ values are reported as inverse serum dilutions. Values of <20 and <10 indicates <50% neutralization at the lowest dilution tested, 1:20 and 1:10 respectively. Viruses used were DENV1 strain WestPac74, DENV2 strain 16803, DENV3 infectious clone 3001,^1^ DENV4 infectious clone,^2, 3^ and Zika virus strain PRVACB59. Years post last infection are calculated from dates of participant self-reported symptoms.

References

1.	Messer WB, Yount B, Hacker KE, Donaldson EF, Huynh JP, de Silva AM, Baric RS. Development and characterization of a reverse genetic system for studying dengue virus serotype 3 strain variation and neutralization. PLoS neglected tropical diseases. 2012;6(2):e1486. doi: 10.1371/journal.pntd.0001486 [doi].

2.	Widman DG, Young E, Nivarthi U, Swanstrom JA, Royal SR, Yount BL, Debbink K, Begley M, Marcet S, Durbin A, de Silva AM, Messer WB, Baric RS. Transplantation of a quaternary structure neutralizing antibody epitope from dengue virus serotype 3 into serotype 4. Sci Rep. 2017;7(1):17169. doi: 10.1038/s41598-017-17355-5. PubMed PMID: 29215033; PMCID: PMC5719398.

3.	Gallichotte EN, Widman DG, Yount BL, Wahala WM, Durbin A, Whitehead S, Sariol CA, Crowe JE, Jr., de Silva AM, Baric RS. A New Quaternary Structure Epitope on Dengue Virus Serotype 2 Is the Target of Durable Type-Specific Neutralizing Antibodies. mBio. 2015;6(5):10.1128/mBio.01461-15. doi: 10.1128/mBio.01461-15 [doi].
